# Instrumental Evaluation of the Effects of Vertebral Consolidation Surgery on Trunk Muscle Activations and Co-Activations in Patients with Multiple Myeloma: Preliminary Results

**DOI:** 10.3390/s24113527

**Published:** 2024-05-30

**Authors:** Barbara Montante, Benedetta Zampa, Luca Balestreri, Rosanna Ciancia, Giorgia Chini, Alberto Ranavolo, Maurizio Rupolo, Zimi Sawacha, Martina Urbani, Tiwana Varrecchia, Mariagrazia Michieli

**Affiliations:** 1 Unit of Onco-Hematology and Stem Cell Transplantation and Cellular Therapies, Centro di Riferimento Oncologico di Aviano (CRO), Istituto di Ricovero e Cura a Carattere Scientifico, 33081 Aviano, Italy; barbara.montante@cro.it (B.M.); benedetta.zampa@cro.it (B.Z.); rciancia@cro.it (R.C.); mrupolo@cro.it (M.R.); mmichieli@cro.it (M.M.); 2 Radiology Department, Centro di Riferimento Oncologico di Aviano (CRO), Istituto di Ricovero e Cura a Carattere Scientifico, 33081 Aviano, Italy; lbalestreri@cro.it (L.B.); murbani@cro.it (M.U.); 3 Department of Occupational and Environmental Medicine, Epidemiology and Hygiene, Istituto Nazionale per l’Assicurazione Contro gli Infortuni sul Lavoro, 00078 Monte Porzio Catone, Italy; a.ranavolo@inail.it (A.R.); t.varrecchia@inail.it (T.V.); 4 Department of Information Engineering, University of Padua, 35131 Padua, Italy; zimi.sawacha@dei.unipd.it

**Keywords:** vertebral consolidation surgery, trunk muscle activations, trunk muscle co-activations, patients with multiple myeloma

## Abstract

Multiple myeloma (MM) patients complain of pain and stiffness limiting motility. To determine if patients can benefit from vertebroplasty, we assessed muscle activation and co-activation before and after surgery. Five patients with MM and five healthy controls performed sitting-to-standing and lifting tasks. Patients performed the task before and one month after surgery. Surface electromyography (sEMG) was recorded bilaterally over the erector spinae longissimus and rectus abdominis superior muscles to evaluate the trunk muscle activation and co-activation and their mean, maximum, and full width at half maximum were evaluated. Statistical analyses were performed to compare MM patients before and after the surgery, MM and healthy controls and to investigate any correlations between the muscle’s parameters and the severity of pain in patients. The results reveal increased activations and co-activations after vertebroplasty as well as in comparison with healthy controls suggesting how MM patients try to control the trunk before and after vertebroplasty surgery. The findings confirm the beneficial effects of vertebral consolidation on the pain experienced by the patient, despite an overall increase in trunk muscle activation and co-activation. Therefore, it is important to provide patients with rehabilitation treatment early after surgery to facilitate the CNS to correctly stabilize the spine without overloading it with excessive co-activations.

## 1. Introduction

Multiple myeloma (MM) is a hematologic malignancy caused by the clonal proliferation of plasma cells, producing a monoclonal immunoglobulin and determining bone marrow infiltration and bone destruction. The symptoms of the disease, most frequently, may be related to anaemia, renal dysfunction, or clonal plasma cell expansion. Clonal plasma cells proliferate in the bone marrow and can involve extramedullary tissues, resulting in spinal column or radicular lesions, vertebral collapses, kyphosis, deformities, paralysis, neurological symptoms, perceived pain, sensory loss, and symmetrical lower limb weakness [1,2,3,4]. The primary cause of morbidity and mortality in patients with multiple myeloma is bone damage, which puts people at risk for physical decline [5].

MM’s clinical features and the effects they have on motor function necessitate thorough patient care regarding every day and occupational activities including getting out of a chair and lifting objects [4,6]. In the 7.8% of patients with MM who have vertebral collapses, vertebroplasty surgery can be performed to biomechanically stabilize the microfractures, relieve pain, and prevent the spine from experiencing further damage [1,2,4,7]. Among the main surgical procedures, percutaneous vertebroplasty offers patients significant and long-lasting pain alleviation [8,9]. Furthermore, literature studies have shown that this procedure produces recovery from disability with a recovery of mobility and a decrease in analgesic drugs use [10,11].

Anyway, it is still unclear how vertebroplasty induces modifications on the behavior of the primary trunk muscles during the execution of daily life activities. In particular, the lack of information on the changes in patients’ activation and co-activation in response to the surgery and the possible pain reduction represents a current scientific literature gap. Muscle co-activation is the simultaneous contraction of a muscle and its antagonist. The central nervous system (CNS) controls muscle co-activation to complete precise tasks, learn new movements, engage in “fear-avoidance” behaviour, and stabilize or protect the joint [12,13,14,15,16]. Excessive co-activation might be detrimental since it reduces the net joint moment and power required to carry out a specific motor activity and increases joint compressive and shear forces, metabolic costs, and cartilage degeneration [17,18,19,20,21,22,23]. In individuals with MM, the presence of vertebral collapses before the vertebroplasty, the altered biomechanics and mobility of the spine, and the presence of pain before and after vertebroplasty are the leading factors that may determine an enhanced co-activation. Excessive co-activation increases compressive loading across the spine joints which in turn increases the risk of low back disorders before and after the vertebral consolidation surgery. Thus, if co-activation is ignored, the extent of spine loading may be underestimated [17,18,19,20,21,22,23].

To the best of our knowledge, there are no studies in the literature analyzing activations and co-activations of the spine muscles in patients who underwent vertebroplasty surgery during daily living and work activities. Furthermore, there is currently adequate evidence in the scientific literature that sEMG can be a beneficial tool for monitoring patients’ development before and after specific types of rehabilitation treatments and/or procedures [24,25,26,27]. Indeed, a well-designed rehabilitation program/clinical monitoring should consider several current therapeutic developments. One of the instrumental-based methods used to assess the efficiency of rehabilitation/surgical therapies is sEMG, which is a multi-channel, non-invasive, wireless, wearable tool that permits in-depth investigation of motor coordination mechanisms [24,25,26,27]. Knowledge of their behaviors would allow for the appropriate referral of these patients to a specific rehabilitation program.

The aim of this study was to compare both activations and co-activation of the trunk muscles during sit-to-stand and lifting activities before and after vertebroplasty surgery. We chose these two activities because they are frequently performed in everyday and occupational life [28,29]. A further aim was to monitor the pain perception of patients with MM and correlate it with muscle behaviors.

We hypothesized that trunk muscle activations, as well as co-activation, will increase following vertebroplasty surgery, and we also assume that the patient experiences advantages mostly on the side of pain.

In the case that the study’s findings support this notion, it would be critical to plan early rehabilitation programs for these individuals with the goal of reducing the strains exerted on the spine’s joints because of muscular co-activation.

## 2. Materials and Methods

### 2.1. Subjects

A convenient sample of 8 patients (3 females and 5 males; mean age 65.88 ± 7.18 years; Body Mass Index (BMI) 27.53 ± 2.70 kg/m^2^) with MM from the Oncology Reference Centre (CRO) of Aviano and 5 healthy controls (HC) (4 females and 1 male; mean age 31.40 ± 7.13 years; BMI 22.19 ± 2.79 kg/m^2^) were enrolled. Before taking part in the study, we confirmed that the study’s inclusion criteria were met by the patients using a standardized questionnaire. The following eligibility requirements were used:

- age ≥ 18 years old;
- BMI < 28 kg/m^2^;
- historically confirmed diagnosis of MM accompanied by multiple vertebral lesions;
- clinical indication and eligibility to perform vertebroplasty procedure;
- performance status (ECOG) 0–2;
- life expectancy greater than three months at the time of enrolment;
- TC skeleton in its entirety low resolution at disease onset and/or follow-up;
- presence of spine pain with stiffness and functional impediment before vertebroplasty;
- absence of risk of spinal cord injury;
- no unstable vertebral injuries requiring orthopedic bracing;
- able to express appropriate consent to participation.

Based on these inclusion criteria, three out of the eight patients were excluded because they were not able to perform the post vertebroplasty recordings while the remaining five patients took part in the study (2 females and 3 males; mean age 63.20 ± 7.85 years; Body Mass Index (BMI), 29.17 ± 2.42 kg/m^2^). 

The ethical aspects of this clinical trial follow the guidelines of the Declaration of Helsinki and all participants signed an informed consent form after receiving a full explanation of the study procedure. This study was approved by the local ethics committee: CEUR-FVG (Single Regional Ethics Committee) under approval number: CRO-2020-35. No information regarding the expected results was provided to avoid the results being biased, whether consciously or unconsciously. 

### 2.2. Pain Assessment

In order to assess the severity of pain before and after the vertebroplasty and its impact on functioning we used the Brief Pain Inventory (BPI), a scale to measure and assess the pain in cancer patients [30]. The BPI is composed by the following pain and interference items: worst pain in last 24 h, least pain in last 24 h, pain on average, pain right now, treatments or medications received for the pain, relief received from pain treatments or medication, interference with general activity, mood, walking ability, normal work including housework, relations with other people, sleep, and enjoyment of life. 

### 2.3. Instrumental Measurements

Kinematic data were collected by using a five-infrared-camera optoelectronic motion analysis system at a sampling frequency of 340 Hz (SMART-DX 6000 System, BTS, Milan, Italy). In detail, we investigated the kinematic of the anatomical landmark of acromion [31] in the sit-to-stand task, while a maker applied the weight in the lifting task. The sEMG signals were recorded at a sampling rate of 1 kHz using an 8-channels bipolar wireless system (FreeEMG 1000 System, BTS, Milan, Italy). After skin preparation, four Ag/AgCl bipolar electrodes precoated with electroconductive gel (2 cm diameter; H124SG Kendall ARBO, Tyco Healthcare, Dublin, Ireland) were placed bilaterally over the erector spinae (ES) longissimus (denoted in the following as RESL and LRESL, respectively, for right and left ES longissimus) and the rectus abdominis (RA) superior (denoted in the following as RRAS and LRAS, respectively, for right and left RA superior) placed with the orientation parallel to the muscle fibers where specified for these muscles according to the European Recommendation for Surface Electromyography (SENIAM) [32], and the atlas of muscle innervation zones [33]. In particular, the electrodes on RESL and LESL were placed vertically at 2 finger-width laterals from the process spinous of L1 and those on RRAS and LRAS between 0% and 13% of the line on the right portion of the muscle belly parallel to the line that starts at the level of the xiphoid process and ends at the level of the superior anterior iliac spine. The acquisition of kinematic, kinetic, and sEMG data was synchronized.

### 2.4. Experimental Procedure

**Figure 1:**
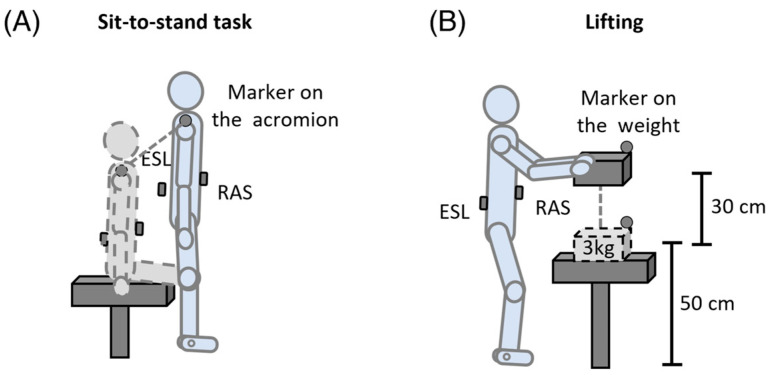
Experimental setup description of the sit-to-stand (**A**) and lifting (**B**) tasks. ESL: erector spinae longissimus; RAS: rectus abdominis superior.

Before the recording session, patients and controls practiced for a few minutes to familiarize themselves with the procedure. All the participants were asked to perform two consecutive motor tasks: sit-to-stand and lifting. For each motor activity, five trials per patient and five trials per healthy subject were recorded. The order of each motor activity was randomly assigned and separated by a 10 min rest to avoid muscle fatigue. More in detail, as far as sit-to-stand was concerned, all the participants were asked to start the task in a seated position with their arms along the body and then to stand up without providing instructions on the motor execution strategy (Figure 1A). While in regards to the lifting task, patients and healthy subjects were instructed to lift a 3 kg weight, placed at an initial height from the ground of 50 cm with a vertical displacement of 30 cm (Figure 1B). Only the signals recorded during the lifting phase were analyzed. 

### 2.5. Data Processing

Data processing was performed using BTS SMART Tracker and Analyzer (version 1.10, BTS Bioengineering, Milan, Italy) and Matlab (version 2021a 9.10.0.2015706, MathWorks, Natick, MA, USA). 

#### 2.5.1. sEMG Signal Processing

Raw sEMG signals were processed with a band pass Hamming filter (30–400 Hz cut-off frequency) in order to reduce motion artifacts and other components of high frequency noise and to remove the ECG artifacts from all the trunk muscles [34,35]. Then, we filtered the signals with a Notch’s filter with cut-off frequency of 50 Hz, to remove noise due to power signal. Then the signals were rectified and 5 Hz low-pass Hamming-filtered to obtain the envelope. Each muscle’s signal was then amplitude-normalized, to be expressed as a percentage of the sEMG peak. More precisely, we normalized the sEMG amplitude to a range of 0–100% by dividing the instantaneous amplitude by the sEMG peak value, calculated as the maximum value of each trial of a participant’s motor activity [36].

#### 2.5.2. Events Identification in the Sit-to-Stand and Lifting Tasks

Start and stop events were defined for each performed task and the corresponding sEMG amplitude-normalized signals were time-normalized between the two events and reduced to 101 samples using a polynomial procedure. Particularly, we used a linear interpolation to curve fitting using linear polynomials to construct new data points within the range of a discrete set of known data points. The time-normalization was used to compare tasks of different durations, and it was referred to as 101 percent samples (where sample 1 is 0% of the cycle and sample 101 is 100%) [36,37,38]. 

In particular, start and stop events of each sit-to-stand task were determined considering the vertical position of the markers placed on the right and left acromions using a self-developed software (BTS Smart Analyzer, BTS, Milan, Italy). The marker’s position was low-pass filtered (4 Hz, Butterworth 4th order). The person was sat on the chair with his/her back straight and the beginning of the task was selected as the instant before the person bends his/her torso forward in the attempt to stand up. The stop event corresponds to the instant when the subject was completely standing, and the position of the shoulder markers were at their higher point in relation to the ground throughout the execution of the task.

Similarly, start and stop events of each lifting were determined considering the vertical velocity of one of the markers placed on the weight using a self-developed software (BTS Smart Analyzer, BTS, Milan, Italy). Before the event’s definition, the marker’s velocity was determined from its position, after a 4 Hz low-pass filter (Butterworth 4th order), by calculating finite difference derivatives, then the velocity was low-pass filtered (4 Hz, Butterworth 4th order). The start corresponded to the time instant when the vertical component of the velocity of the marker on the weight exceeded the limit value of 0.025 m/s, while the stop corresponded to the instant when it falls below the limit value in the opposite direction [37,38].

#### 2.5.3. Co-Activation Function 

Both for sit-to-stand and lifting tasks, for each trial executed by the subjects, the simultaneous activation of the two trunk antagonist muscle groups, co-activation function, was calculated by applying the following formula [39]:

(1) $$C\left(i\right)=\left[{EMG}_{H}\left(i\right)+{EMG}_{L}\left(i\right)\right]\times \left[{EMG}_{L}\left(i\right)/{EMG}_{H}\left(i\right)\right]$$

where i is the *i*-th sample of the sEMG signals, and ${EMG}_{H}$ and ${EMG}_{L}$ are the highest and the lowest activity between the antagonist muscle pairs (ESL and RAS). This method provided a sample-by-sample estimate of the relative activation of the pair of muscles as well as the magnitude of the co-activation over the entire cycle. With the use of this equation, high co-activation values represent a high level of activation of both muscles across a large time interval, whereas low co-activation values indicate either low level activation in both muscles or a high level activation in one muscle along with low level activation in the other muscle in the pair [39]. To characterize differences in co-activation amplitude, we chose as a punctual co-activation index (CI), the maximum (${CI}_{Max}$), which provides instantaneous information about the peak at which the co-activation arrives, and an extended index, the mean (${CI}_{Mean}$), which expresses what happens on average throughout the entire task. ${CI}_{Max}$ and ${CI}_{Mean}$ values within the lifting cycles were calculated as follows:

(2) $${CI}_{Max}={max}_{1,\cdots ,n}\left(C\left(i\right)\right)$$

(3) $${CI}_{Mean}=\frac{1}{n}\sum_{i=1}^{n}C\left(i\right)$$

where n is the length of the signal (101 samples in this case), and $CI\left(i\right)$ is the *i*-th sample of the Rudolph’s co-activation function. Furthermore, to characterize the timing of the co-activation function, we computed the full width at half maximum (*FWHM*) of the co-activation function (${CI}_{FWHM}$), which provides the sum of the durations of all the time intervals throughout each task when the co-activation surpassed half of its maximum, according to the following formula:

(4) $${CI}_{FWHM}=\sum_{j}{\Delta t}_{j}$$

where ${\Delta t}_{j}$ is the duration of the *j*-th interval in which the co-activation function is above half of its maximum. 

#### 2.5.4. Trunk Muscles Parameters

Both for the sit-to-stand and lifting tasks, for each trial executed by each subject, to evaluate the muscle activation, we calculated the same synthetic indexes described above for the co-activation function (mean, maximum, and FWHM) for each of the four investigated trunk muscles (${RESL}_{Mean}$, ${RESL}_{Max}$, ${RESL}_{FWHM}$; ${LESL}_{Mean}$, ${LESL}_{Max}$, ${LESL}_{FWHM}$; ${RRAS}_{Mean}$, ${RRAS}_{Max}$, ${RRAS}_{FWHM}$; ${LRAS}_{Mean}$, ${LRAS}_{Max}$, ${LRAS}_{FWHM}$). Furthermore, using the band-pass filtered sEMG data recorded during tasks, we calculated the power spectral density using Yule–Walker’s approach (the autoregressive parameters were estimated using Levinson Durbin recursion with a model order 15) [40], and its median (MDF) and mean frequency (MNF) were computed for each muscle (${RESL}_{MDF}$, ${RESL}_{MNF}$; ${LESL}_{MDF}$, ${LESL}_{MNF}$; ${RRAS}_{MDF}$, ${RRAS}_{MNF}$; ${LRAS}_{MDF}$, ${LRAS}_{MNF}$).

### 2.6. Statistical Analysis

The statistical analysis was performed using SPSS 20.0 (IBM SPSS). For each subject and each task (sit-to-stand and lifting), the data of all the trials were averaged. Preliminarily, the Shapiro–Wilk normality test was applied to check for normal distribution. Depending on the results of the normality test, the independent-samples t-test or the Mann–Whitney test was used to detect any significant differences in the co-activation and trunk muscles parameters (${CI}_{Mean}$, ${CI}_{Max}$, ${CI}_{FWHM}$; ${RESL}_{Mean}$, ${RESL}_{Max}$, ${RESL}_{FWHM}$; ${LESL}_{Mean}$, ${LESL}_{Max}$, ${LESL}_{FWHM}$; ${RRAS}_{Mean}$, ${RRAS}_{Max}$, ${RRAS}_{FWHM}$; ${LRAS}_{Mean}$, ${LRAS}_{Max}$, ${LRAS}_{FWHM}$; ${RESL}_{MDF}$, ${RESL}_{MNF}$; ${LESL}_{MDF}$, ${LESL}_{MNF}$; ${RRAS}_{MDF}$, ${RRAS}_{MNF}$; ${LRAS}_{MDF}$, ${LRAS}_{MNF}$) between the healthy controls and the patients before and after the vertebroplasty surgery, while the paired t-test or the Wilcoxon test was used to identify any major differences in the co-activation and trunk muscle parameters in patients with MM before and after the vertebroplasty and in the BPI.

The Pearson’s test was used to investigate any correlations between the co-activation and trunk muscles parameters and the BPI items in patients with MM before and after the surgery. The significance level for all statistical analyses was set at *p*-value <0.05.

## 3. Results

### 3.1. Pain Assessment Results

Table 1 shows the pain scores (0–10) before and after the vertebroplasty surgery. The statistical analysis revealed a significant decrease in the “pain inst” (*p* = 0.032) and “How, during the past 24 h, pain has interfered with: Relations with other people” (*p* = 0.032).

### 3.2. sEMG Parameters Results

**Figure 2:**
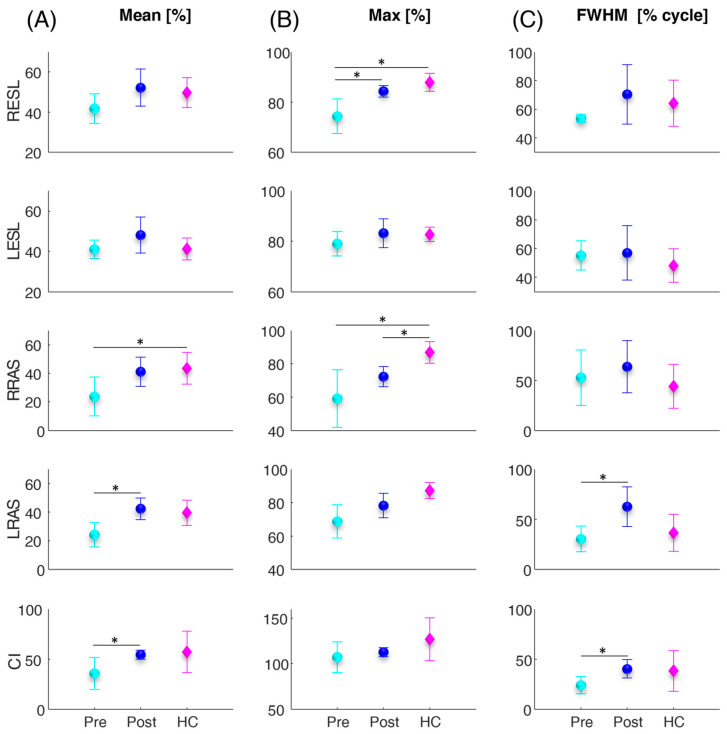
Average (circles and diamonds) and standard deviation (vertical lines) values over all subjects of the mean (**A**), maximum (max) (**B**), and full width at half maximum (FWHM) (**C**), of the four investigated trunk muscles (RESL, LESL, RRAS, LRAS) and of the co-activation function (CI) for the patients before and after the vertebroplasty surgery (cyan and blue filled circles denoted as Pre and Post, respectively) and for the healthy controls (magenta filled diamonds denoted as HC) computed during the sit-to-stand task. The asterisks (*) indicate significant differences.

Figure 2 shows the error bar and statistical (asterisks) results related to the trunk muscles and co-activation parameters calculated for the sit-to-stand task. Between the pre and post vertebroplasty, the statistical analysis revealed a significant increase in the ${LRAS}_{Mean}$ (*p* = 0.008), ${CI}_{Mean}$ (*p* = 0.034), ${RESL}_{Max}$ (*p* = 0.015), ${LRAS}_{FWHM}$ (*p* = 0.015), and ${CI}_{FWHM}$ (*p* = 0.020). From the comparison between the control subjects and the patients, before and after the vertebroplasty intervention, the statistical analysis highlighted in the patients, before the intervention, statistically significantly lower values compared with the controls in terms of the ${RRAS}_{Mean}$ (*p* = 0.036), ${RESL}_{Max}$ (*p* = 0.004), and ${RRAS}_{Max}$ (*p* = 0.010), and statistically significantly lower values in patients after the surgery, compared with the controls in terms of the ${RRAS}_{Max}$ (*p* = 0.006). 

Mean (circles and diamonds) and standard deviation (vertical lines) values over all subjects of the mean (A), maximum (max), and full width at half maximum (FWHM) of the four investigated trunk muscles (RESL, LESL, RRAS, LRAS) and of the co-activation function (CI) for the patients before and after the vertebroplasty surgery (cyan and blue filled circles denoted as Pre and Post, respectively) and for the healthy controls (magenta filled diamonds denoted as HC) computed during the sit-to-stand task. The asterisks (*) indicate significant differences.

**Figure 3:**
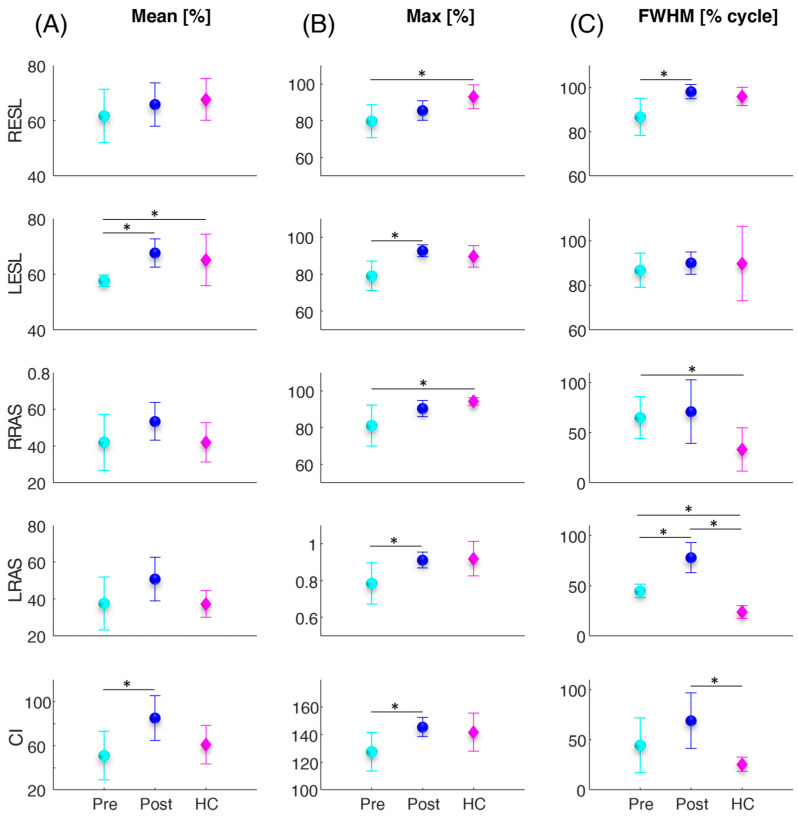
Average (circles and diamonds) and standard deviation (vertical lines) values over all subjects of the mean (**A**), maximum (max) (**B**), and full width at half maximum (FWHM) (**C**) of the four investigated trunk muscles (RESL, LESL, RRAS, LRAS) and of the co-activation function (CI) for the patients before and after the vertebroplasty surgery (cyan and blue filled circles denoted as Pre and Post, respectively) and for the healthy controls (magenta filled diamonds denoted ad HC) computed during the lifting task. The asterisks (*) indicate significant differences.

Figure 3 illustrates the error bar and statistical (asterisks) results related to the trunk muscles and co-activation parameters computed for the lifting task. Between the pre and post vertebroplasty, the statistical analysis revealed a significant increase in the ${LESL}_{Mean}$ (*p* = 0.003), ${CI}_{Mean}$ (*p* = 0.034), ${LESL}_{Max}$ (*p* = 0.008), ${LRAS}_{Max}$ (*p* = 0.047), ${CI}_{Max}$ (*p* = 0.032), ${RESL}_{FWHM}\;$(*p* = 0.022), and ${LRAS}_{FWHM}$ (*p* = 0.002). From the comparison between the control subjects and the patients, before and after the vertebroplasty intervention, the statistical analysis highlighted in the patients, before the intervention, statistically significantly lower values compared to the controls in terms of the ${LESL}_{Max}$ (*p* = 0.043), ${RESL}_{Max}$ (*p* = 0.027), and ${RRAS}_{Max}$ (*p* = 0.031), and significant higher values in patients with respect to the controls before the surgery in the ${RRAS}_{FWHM}\;$(*p* = 0.046) and ${LRAS}_{FWHM}$ (*p* = 0.001) and after the surgery in the ${LRAS}_{FWHM}$ (*p* < 0.001), and ${CI}_{FWHM}$ (*p* = 0.009).

**Figure 4:**
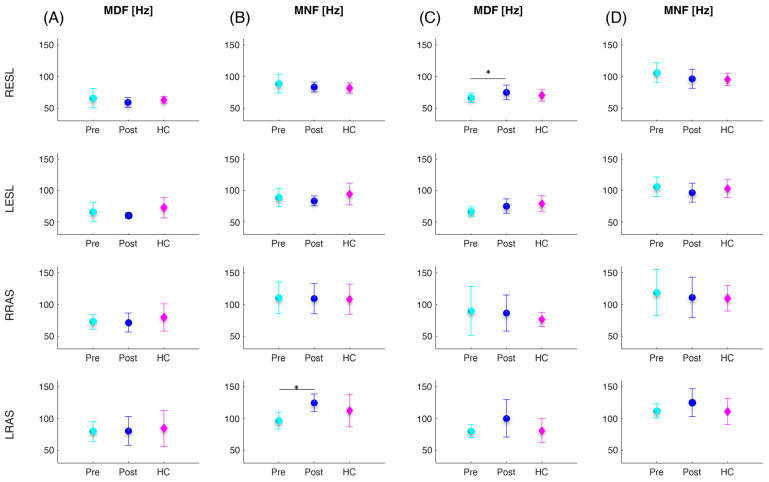
Mean (circles and diamonds) and standard deviation (vertical lines) values over all subjects of the median (MDF) and mean (MNF) frequency of the four investigated trunk muscles (RESL, LESL, RRAS, LRAS) for the patients before and after the vertebroplasty surgery (cyan and blue filled circles denoted as Pre and Post, respectively) and for the healthy controls (magenta filled diamonds denoted ad HC) computed during the sit-to-stand (**A**,**B**) and lifting (**C**,**D**) tasks. The asterisks (*) indicate significant differences.

Figure 4 shows the error bar and statistical (asterisks) results related to the trunk muscles’ frequency parameters calculated for the sit-to-stand (Figure 4A,B) and lifting (Figure 4C,D) tasks. Between the pre and post vertebroplasty, the statistical analysis revealed a significant increase in the ${LRAS}_{MNF}$ (*p* = 0.012) in the sit-to-stand task and ${RESL}_{MDF}$ (*p* = 0.010) in the lifting task. From the comparison between the control subjects and the patients, no statistically significant differences were found.

### 3.3. Correlations

**Figure 5:**
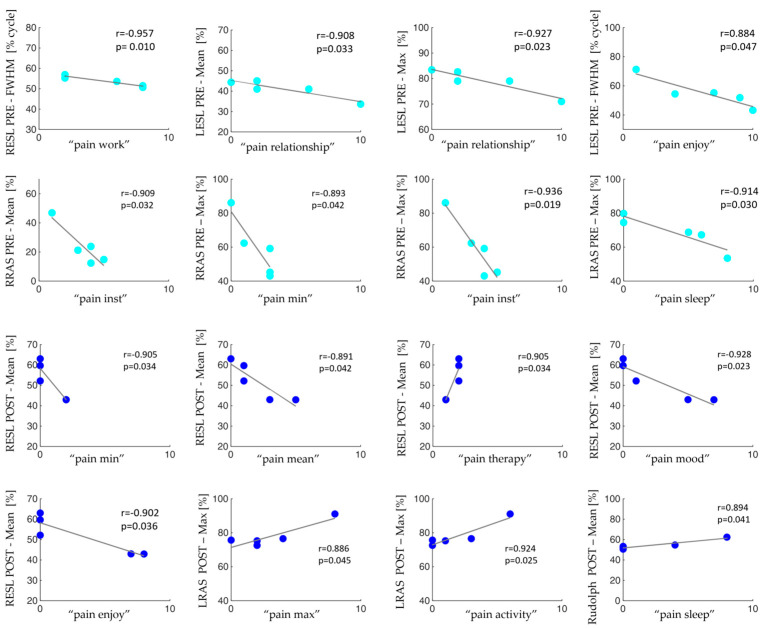
Data scatter plots and the fit line together with the Pearson’s coefficient (r) and the statistically significant *p* values between co-activation and trunk muscle parameters and the BPI items in patients with MM before (cyan dots) and after (blue dots) the surgery in the sit-to-stand activity.

Figure 5 illustrates the statistically significant correlations between co-activation and trunk muscle parameters and the BPI items in patients with MM before and after the surgery in the sit-to-stand activity.

**Figure 6:**
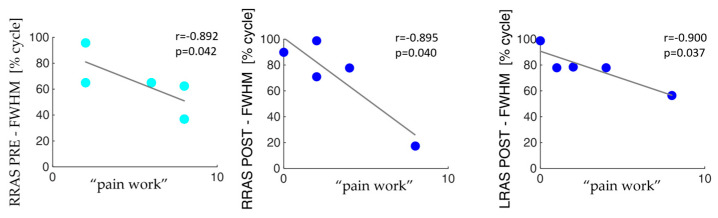
Data scatter plots and the fit line together with the Pearson’s coefficient (r) and the statistically significance *p* values between co-activation and trunk muscles parameters and the BPI items in patients with MM before (cyan dots) and after (blue dots) the surgery in the lifting activity.

Figure 6 shows the corresponding results related to the lifting activity.

## 4. Discussion

This work aimed to investigate the effects of vertebroplasty surgery on trunk muscle activity and co-activation and pain severity in patients with MM. The final objective was to understand how the spine is stressed even after vertebroplasty surgery in order to better define any following rehabilitation treatment. 

In more detail, both for the sit-to-stand and lifting activities, we found an overall increase in all muscle activity parameters (average, maximum, and FWHM) for all four trunk muscles investigated (see Figure 2 and Figure 3). In particular, a consistent trend was also found for muscle co-activation calculated with the Rudolph equation [39]. Indeed, after the vertebroplasty, the average, maximum, and FWHM of the Rudolph co-activation function increased, and this increment was statistically significant, both in the sit-to-stand activity and in the lifting (see Figure 2 and Figure 3). Regarding the frequency parameter results, an increase in the mean frequency of the left rectus abdominis (see Figure 4B) and in the median frequency of the right erector spinae (see Figure 4C) were highlighted one month after the vertebroplasty compared with the parameters measured before it.

These findings may lead to different considerations: First, in the pre-vertebroplasty state, the CNS places the commitment of the muscles in a minimum state with the aim of not overloading the spine despite the presence of pain. Naturally, in this condition, the ability to stabilize the structure is minimal. At the time of post vertebroplasty surgery, the increase in activations and co-activations indicates that the CNS is able to better stabilize the structure of the spine more similarly to the controls. Indeed, the comparison with healthy controls showed that the patients’ muscular behavior was less similar to one of the controls before the spinal consolidation procedure, while the post-operative condition resembled more closely the behavior of the controls (see Figure 2 and Figure 3). Conversely, although the pain decreases, in some cases after the surgery (see ${CI}_{FWHM}$), co-activation may exceed the numbers recorded in the control subjects, and this can lead to some disadvantages. It has been demonstrated that co-activation increases the compressive forces on the spine [36,37,41,42] and this can be detrimental to the structure, already “burdened” by the surgery. Increased co-activation and activity of the ESL and RAS antagonist muscles for more than fifty percent of the time spent performing the task results in increased trunk stiffness [12,13]. Trunk stiffness is a major contributor to spinal stability. Stability describes the ability to maintain balance in the presence of kinematic and/or control impairments. Muscle co-contraction contributes to trunk stiffness because stiffness is mostly connected with active muscles in the trunk musculature rather than passive tissues [12]. Furthermore, the scientific scenario indicates that co-activation contributes to a reduction in net moments at the L5-S1 joint and a 12–18% increase in spinal load, which in turn leads to an increased risk of low-back disorders (LBDs) [17,36]. Therefore, in the post vertebroplasty condition, the lumbar spine is subjected to higher compressive and shear forces, which imply a greater risk of injury [17]. Furthermore, the increasing mean frequency of the left rectus abdominis (see Figure 4B) and in the median frequency of the right erector spinae (see Figure 4C) one month after the vertebroplasty could be associated with a reduction in fatigue. However, different studies have shown how the results in the spectral domain are not homogenous [43,44]. In general, the frequency shift has been attributed to changes in the recruitment and synchronization of the motor units [44]; it also identifies the type of motor units recruited [45] and the type of fiber [46], and is also more sensitive to the diameter of the fibers [44]. Furthermore, in the frequency domain, the median and the mean frequency show better performance than other domain features in the case of assessing muscle fatigue [47]. However, in dynamic movements, to obtain robust results, more attention should be paid to the effects of muscle strength and muscle geometry [43] when the task is performed, as they affect MNF and MDF values [43,44]. Therefore, these results should be further investigated.

The post vertebroplasty period is therefore a very delicate phase in which this increased co-activation needs to be counteracted from the outset by means of appropriate trunk-centered rehabilitation treatment such as Progressive Modular Rebalancing [48]. In the literature, it has been shown that these treatments, in addition to an improvement in the gait spatiotemporal parameters and joint kinematics, contribute to a significant improvement in trunk muscle coordination [48]. Because the head and trunk represent approximately 60% of the total body mass, the ability to accurately coordinate trunk movements during daily life activities is critical in establishing a more energy-efficient movement by connecting the action of the trunk and pelvis like a resonant pendulum, while lowering the whole’s momentum [49].

Unfortunately, as stated in the introduction, there is no scientific literature on the evaluation of the kinematic behaviour of the trunk and its muscles in MM patients. However, the findings are comparable with what is reported in the literature in other studies where sEMG is used to evaluate the impact of surgery in patients with vertebral instability [50,51]. The results of these studies reveal that trunk muscle activity changes following surgery. In particular, these studies show that it is the erector spinae muscle that is most and for a longer time engaged following the surgery. Furthermore, a further study shows that in adult patients with spinal deformity there is greater trunk muscle activity when standing in the erect position than in healthy controls, which is congruent with the findings of the current investigation [52]. This comparison with the literature, on the one hand, confirms the validity of sEMG for assessing the effect of surgery in a variety of patients, from neurological to spinal deformity to oncological diseases, and preventing impaired spine mobility and muscle activation. On the other hand, the increased trunk muscular activations at one month after surgery indicate the necessity to expose patients to specific trunk rehabilitation so that they recover as much as possible to normality following surgery, which has already been established at three months after surgery for patients with lumbar instability [51], and in disabling occupational lumbar spinal disorder patients with prior work-related injuries in [50].

The correlation analysis highlighted that, in the pre-surgery condition, the pain tends to decrease as the overall activity of the analyzed muscles increases. Additionally, after vertebroplasty, the only activation parameters that show a negative correlation with pain are ${RESL}_{Mean}$, ${RESL}_{FWHM}$, ${RRAS}_{FWHM}$, and ${LRAS}_{FWHM}$ considering both sit-to-stand and lifting. This decrease in the number of negative correlations between the activation of the muscles of interest and the pain interfering items may reflect the stabilization process achieved by vertebral consolidation surgery. 

One notable drawback of this study is the small number of patients recruited in the study sample up to this stage in the experimental phase, which limits the ability to generalize our findings. On the other hand, multiple myeloma is to be considered, among adult neoplasms, a rare pathology in terms of incidence and prevalence. By virtue of these data and the eligibility requirements, in the study period approved by the ethical committee, the number of patients referred to our center and recruited into the study could not be greater. In particular, the recruited patients had to present with bone disease (not always present at the onset and relapse), but not so severe as to imply the use of orthopedic braces for standing. Furthermore, at post vertebroplasty evaluation, patients who had shown significant progression of bone disease were excluded from the study (three out of eight patients were excluded because they were not able to perform the post vertebroplasty recordings). Finally, at any phase in the study, patients could develop iatrogenic neuropathy as a side effect, with motor effects that would overlap with those induced by vertebroplasty, representing a bias. For this reason, the results should be understood as preliminary. As a result, the first possible future development is the extension of the examined sample. A further limitation is represented by the fact that the two samples of patients and controls are not matched for age and gender.

Furthermore, another limitation is caused by the contamination of the electrocardiogram (ECG) affecting the two superior rectus abdominis muscles. This contamination is a consequence of the proximity of the surface electrodes to the heart and the volume conduction properties of human tissue. For the tasks performed in this study, which require modest muscle activation, the amplitudes of the sEMG signals are considerably affected by the ECG artefact [34]. Complete removal of the ECG contribution to the signal is particularly complex due to the overlapping frequency spectrum of sEMG (20–500 Hz) and ECG (0–200 Hz) signals [34]. Since the majority of sEMG power is found between 20 and 200 Hz, while most ECG power falls below 35 Hz, the presence of the ECG can result in an increase in the power of frequencies in the lower region of the sEMG spectrum and distort the amplitude of the sEMG, contaminating the information of interest from the abdominal muscles [35]. The literature shows that in non-fatiguing regimes, such as those proposed in this study, the use of a high cut-off reduces ECG contamination but also leads to a reduction in amplitude and attenuation of the signal of interest, with a consequent loss of information [35]. As mentioned in the sEMG signal processing paragraph, the high pass Hamming filter used has a cut-off frequency of 20 Hz to reduce as much as possible this artifact.

It has already been proven that instrumental methods of movement analysis in orthopedic and trauma surgery disciplines can aid as an extra diagnostic tool to develop therapy plans and assess therapeutic results [53]. Even in the context of the integration/reintegration into work of patients suffering from neurological pathologies or amputees, for example, it has been demonstrated that quantitative biomechanical and physiological indexes are good tools, with the kinematic ones being widely used and the electromyographic ones still little utilized [54]. Yet, it is proven that sEMG is a multi-channel, non-invasive, wireless, wearable tool that permits in-depth investigation of motor control processes, hence sEMG could be an instrument-based method used to measure the efficiency of ergonomic and rehabilitation therapies [24]. This study, as well as shedding light on the control of the trunk implemented by patients suffering from multiple myeloma before and after the vertebroplasty surgery in the activities of lifting and sit-to-stand, can contribute to further enhancing the capacity of using the sEMG wireless sensor network as a sensitive, efficient, and practical instrument to evaluate the effect of interventions (surgical, rehabilitative, …) even in cancer patients.

## 5. Conclusions

The results of this study confirm the beneficial effect of vertebral consolidation on the pain experienced by the patient, as claimed by the current scientific scenario, despite an overall increase in trunk muscle activation and co-activation. It will be important to provide rehabilitation interventions aimed at facilitating the CNS to correctly stabilize the spine without overloading it with additional compressive forces due to excessive co-activations.

## Figures and Tables

**Table 1:** Values (0–10) of the BPI’s different items (mean ± standard deviation) before (pre) and after the vertebroplasty surgery (post). Statistically significant differences are in bold.

Items	Abbreviation	Pre	Post
Pain at its worst in the last 24 h	“pain max”	5.8 ± 2.3	3.2 ± 2.7
Pain at its least in the last 24 h	“pain min”	2.0 ± 1.3	0.8 ± 0.9
Pain on the average	“pain mean”	4.4 ± 2.2	2.0 ± 1.8
Pain right now	“pain inst”	**3.4 ± 1.4**	**0.8 ± 0.7**
Are you assuming a therapy for the pain? (yes: 1, no: 2)	“pain therapy”	1.2 ± 0.4	1.6 ± 0.5
How, during the past 24 h, pain has interfered with: General Activity	“pain activity”	4.8 ± 2.0	2 ± 2.3
How, during the past 24 h, pain has interfered with: Mood	“pain mood”	2.4 ± 2.5	2.6 ± 2.9
How, during the past 24 h, pain has interfered with: Walking ability	“pain walking”	6.2 ± 2.5	2.4 ± 3.0
How, during the past 24 h, pain has interfered with: Normal Work (includes both work outside the home and housework)	“pain work”	5.2 ± 2.7	3.0 ± 2.8
How, during the past 24 h, pain has interfered with: Relations with other people	“pain relationship”	**4.0 ± 3.6**	**0.0 ± 0.0**
How, during the past 24 h, pain has interfered with: Sleep	“pain sleep”	3.8 ± 3.2	2.4 ± 3.2
How, during the past 24 h, pain has interfered with: Enjoyment of life	“pain enjoy”	6.2 ± 3.3	3 ± 3.7

## Data Availability

Dataset available on request from the authors.
